# Exome sequencing identified novel variants in three Chinese patients with 5,10-methenyltetrahydrofolate synthetase deficiency

**DOI:** 10.3389/fgene.2023.1236849

**Published:** 2023-09-18

**Authors:** Xiaoyan Xu, Jing Zhu, Liwei Fang, Zhuo Zou, Jingjing Yuan, Min Peng, Guoliang Yu, De Wu, Yun Liu, Jiulai Tang

**Affiliations:** ^1^ Pediatric Neurorehabilitation Center, Pediatric Department, The First Affiliated Hospital of Anhui Medical University, Hefei, China; ^2^ Department of Rehabilitation, Kunming Children’s Hospital, Kunming Medical University, Kunming, Yunnan, China; ^3^ Chigene (Beijing) Translational Medical Research Center Co, Ltd, Beijing, China

**Keywords:** 5, 10-methenyltetrahydrofolate synthetase, MTHFS deficiency, folate metabolism, seizure, developmental delay

## Abstract

5,10-methenyltetrahydrofolate synthetase (MTHFS) deficiency is a folate metabolism disorder known as a rare autosomal recessive neurodevelopmental disorder (MIM: #618367). With central nervous system involvements, it is mainly characterized by developmental delay, epilepsy, microcephaly, hypertonia, and cranial nerves involvement. Here, we report three new cases with MTHFS deficiency from two non-consanguineous Chinese families. All patients showed white matter dysplasia and global developmental delay, of which only patient 1 and 2 manifested tonic-clonic seizures. Moreover, patient 2 had severe eczema and patient 3 had recurrent diarrhea. Both phenotypic features are firstly found in MTHFS deficiency. Trio whole-exome sequencing and sanger sequencing were used to identify four novel variants, p.Y169Tfs*17, p.S53F, c.117+1delG, and p.E61G in the MTHFS gene. The identification of four novel pathogenic variants and varied clinical features in three affected patients expands the genotype and phenotype spectrum of MTHFS deficiency. We also reviewed all cases of MTHFS deficiency that had previously been reported. The experience of diagnosis and treatment from these cases provides us a more comprehensive understanding of this rare disease.

## Introduction

The 5,10-methenyltetrahydrofolate synthetase (MTHFS), also known as 5-formyltetrahydrofolate (5-FTHF) cyclo-ligase, is encoded by the MTHFS gene (location: 15q25.1). MTHFS catalyzes the conversion of 5-FTHF to 5,10-MTHF in an ATP and Mg2+ dependent manner, which is subsequently reduced to 5-MTHF and other reduced folates required for neurotransmitter synthesis, amino acid metabolism, and purine/thymidylate biosynthesis (Dayan et al., 1995). MTHFS gene knockdown and overexpression in cell lines or mice indicate that MTHFS regulates cellular folate pools and determines folate levels in tissues, which is crucial for oligodendrocyte maturation and survival in the central nervous system (CNS) (Girgis et al., 1997; Field et al., 2011). MTHFS also potentially affect the translation of mitochondrial methionyl-tRNA by acting as an intra-mitochondrial folate pool regulator in human mitochondria, whose deficiency could contribute to secondary mitochondrial dysfunction (Bertrand et al., 1995; Krupenko et al., 2010). Since folate metabolism and mitochondrial energy metabolism are critical in neural cells and cerebrospinal fluid (CSF), disruption of MTHFS activity can cause a variety of neurological impairments (Zheng and Cantley, 2019).

In recent years, MTHFS deficiency (MIM: #618367) has only been reported in a few unrelated patients as a rare autosomal recessive neurodevelopmental disorder. A total of seven variants in the MTHFS gene were identified in six different cases till now, which results in a range of phenotypes associated with abnormal enzyme activity. The level of 5, 10-MTHF in cerebrospinal fluid is decreased due to MTHFS impairment, whereas peripheral folate levels are normal. The clinical features mainly include global developmental delay (GDD), hypotonia, epilepsy, microcephaly, delayed myelination or hypomyelination and other cerebral dysplasia (Rodan et al., 2018; Romero et al., 2019; Sakthivel et al., 2020; Cavusoglu et al., 2022; Vafaee-Shahi et al., 2022). However, as an extremely rare disorder, there is very limited understanding of this disease.

Here, we present clinical findings of three Chinese patients with MTHFS deficiency, identify four novel variants c.504del (p.Y169Tfs*17), c.158C>T (p.S53F), c.117+1del and c.182A>G (p.E61G) in the MTHFS gene, and review the literature on the clinical features and therapy responses of this disease.

## Materials and methods

### Subjects and study approval

We recruited two families with MTHFS deficiency. Family 1 has two affected siblings and experienced two pregnancies of embryonic demise. The two children were admitted with repetitive seizures to Pediatric Neurorehabilitation Center, The First Affiliated Hospital of Anhui Medical University. The proband of family 2 was referred to the Department of Pediatric Rehabilitation Medicine, Kunming Children’s Hospital due to delayed motor development. This study has been approved by the ethics committee of The First Affiliated Hospital of Anhui Medical University and Kunming Children’s Hospital. Written informed consent for genetic testing and publication of relative information has been obtained from the legal guardians of the children. All investigations followed the principles of the Declaration of Helsinki.

### Genetic tests and analysis

After obtaining prior written consent of the parents, venous blood samples from all patients and their parents were collected and stored properly. Trio whole-exome sequencing (WES) was performed on the genomic DNA extracted from whole blood samples using xGen Exome Research Panel v1.0 (IDT, Iowa, United States) and paired-end sequenced (2 × 150 bp) on NovaSeq 6,000 (Illumina San Diego, CA). Raw sequence readouts were processed by fastp and paired-end sequence reads were aligned to the human GRCh37/hg19 genome using the Burrows-Wheeler Aligner (BWA) and the GATK software packages. Variants annotations were retrieved from the database-based online system independently developed by Chigene (https://www.chigene.org, Beijing, China), including public SNP databases, the Single Nucleotide Polymorphism database (dbSNP), 1000 Genomes Project, Exome Aggregation Consortium (ExAC), Genome Aggregation Database (gnomAD), and ESP, and Chigene in-house MAFs database. Genes and the associated clinical phenotypes in Mendelian disorders were annotated using data from UCSC, RefGene, GENCODE, and ENSEMBL transcripts, LOVD, SWISS, Clinvitae, HGMD, OMIM, and ClinVar databases. Functional and conservational predictions for the amino-acid changes were evaluated by the software packages Provean, SIFT, PolyPhen2, MutationTaster, M-CAP, REVEL, and CADD; MaxEntScan, dbscSNV, and GTAG; GERP, phyloP, and phastCons. Causative variants screening was done according to rare variants with minor allele frequency (MAF) < 0.05% and biological impact, gene functions and disease mechanism, recessive/dominant/*de novo* pattern of inheritance and segregation analysis, phenotypic relevance in Human Phenotype Ontology (HPO) terms and the scientific literature. Subsequently, variants were classified according to the ACMG clinical practice guidelines (Richards et al., 2015). Sanger sequencing was performed on the patients and the parents to validate the candidate variants. The crystal structure of human MTHFS with 10-formyltetrahydrofolate (PDB ID: 3HY3) and disease-associated residues were visualized by Chimera-X.

## Results

### Case description

Family 1

**FIGURE 1:**
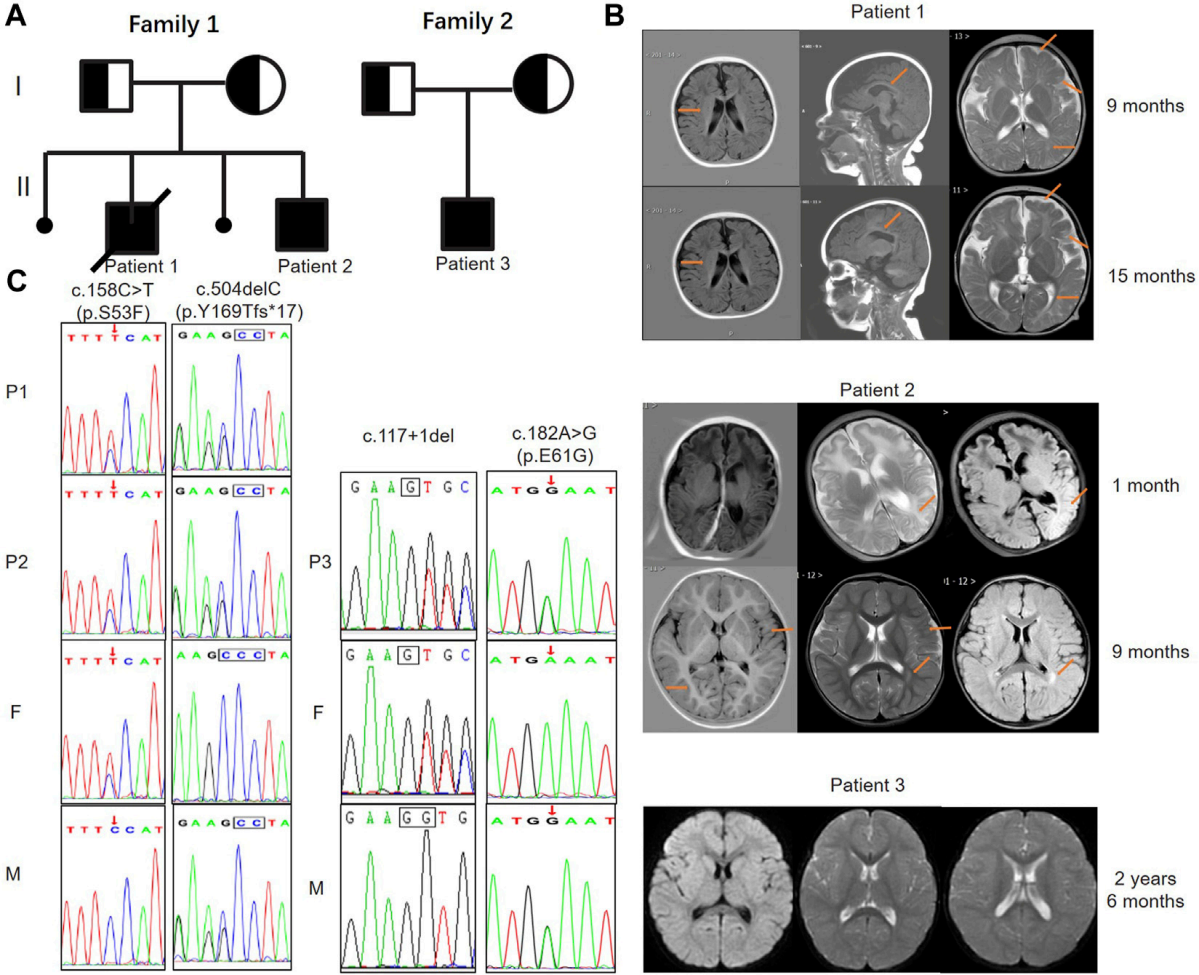
Pedigree diagrams, MRI images, and genetic findings of patients with MTHFS deficiency. **(A)** The pedigree diagrams of family 1 and 2 reveal a recessive inheritance pattern. Family 1 had two pregnancy loss and patient 1 died when he was 22 months old. Patient 3 is the only child in family 2. **(B)** Patient 1: images from left to right are axial T1-weighted, parasagittal T1-weighted, and axial T2-weighted. Widened bilateral frontotemporal subarachnoid, stripe hyperintensity around the lateral ventricle and centrum semiovale, decreased white matter volume, delayed myelination, thin corpus callosum, slightly enlarged lateral ventricles and widened cerebral sulci were observed (arrows) at 9 and 15 months. Patient 2: images from left to right are axial T1-weighted, axial T2-weighted and axial FLAIR. The images at 1 month revealed a slightly higher T2 and FLAIR signal in the left parieto-occipital lobe (arrow). The follow-up images at 9 months showed slightly widened bilateral sulcus fissure of the cortex, widened bilateral frontotemporal subdural space, decreased volume of white matter, and a patchy high T2 and FLAIR signal beside the left lateral ventricle (arrows). Patient 3: images from left to right are axial FLAIR and axial T2-weighted. The images at 2 years and 6 months of age only showed a decrease in the white matter volume of the brain, indicating hypomyelination. **(C)** Sanger sequencing confirms the pathogenic variants and both healthy parents are heterozygous carriers of the MTHFS mutations. Two variants of c.158C>T (p.S53F) and c.504del (p.Y169fs*17) in the MTHFS gene were identified in family 1; c.117+1del and c.182A>G (p.E61G) variants were identified in family 2. F: father; M: mother.

Patient 1 (P1) was a 16-month-old boy admitted to our department after having two seizures at the ages of 15 and 16 months in 2018. He had ocular hypertelorism, long nasolabial fold, and tent-shaped upper lip. Physical examination revealed involuntary movements and hypertonia when excited. He was born from the second pregnancy of a non-consanguineous couple, while the first pregnancy resulted in an embryonic demise, and he was the first child of family 1 (Figure 1A). His mother had hypothyroidism during pregnancy and was treated with levothyroxine. At 6 months of age, he could not actively grasp, and at 9 months of age, he presented an inability to sit without assistance, indicating developmental delay (DD). His cranial MRI at 9 months primarily showed abnormal myelination, widened bilateral frontotemporal subarachnoid, and widened cerebral sulci (Figure 1B).

At 15 and 16 months of age, he respectively experienced frequent seizures after two fever episodes (including partial seizures, tonic seizures, tonic-clonic seizures, and atypical absence seizures), lasting between minutes to hours (status epilepticus). Hypertonia and athetosis were also observed during seizures. Phenobarbital was administered intravenously for sedation. His cranial MRI at 15 months re-demonstrated white matter dysplasia (Figure 1B). Blood routine examination showed mild anemia with low hemoglobin (Hb) of 104 g/L and low hematocrit (HCT) of 31.30%. Serum vitamin B12, lactate (LAC), peripheral folate, fasting blood glucose (FBG), thyroid function, and urine metabolism were normal. Diagnosed with epilepsy, cerebral palsy and GDD, he was treated with oxcarbazepine for anti-seizure therapy and had regular rehabilitation training.

After orally taken oxcarbazepine for 1 month, he developed multiple afebrile convulsions. Then the anti-seizure therapy regimen was replaced with sodium valproate and lamotrigine, but he still had petit mal seizures. At the age of 20 months, he had short stature, could not sit without assistance and pronounced unconsciously, indicating GDD. His cognitive, motor and speech functions did not improve after rehabilitation training. Unfortunately, the patient died from sudden unexpected death of epilepsy (SUDEP) at 22 months of age.

Patient 2 (P2), male, 3 months old, was admitted to our department due to recurrent seizures in 2022. He had craniofacial dysmorphisms including microcephaly, oxycephaly, long nasolabial fold, tent-shaped upper lip, and high arched palate. Tapered fingers were observed. Physical examination showed hypertonia, poor head control, poor eye contact and interaction, and severe eczematous dermatitis. He was the second child and was born from the fourth pregnancy of family 1. The third pregnancy of his mother was also ended by an embryonic demise (Figure 1A).

P2 was delivered by cesarean section at term without asphyxia or hypoxia and his Apgar score was 10–10. On the second day after birth, he was hospitalized due to shivering. Electroencephalogram (EEG) was normal and symptomatic treatment such as hyperbaric oxygen therapy was given. At the age of 40 days, he had frequent seizures in 1 day (focal seizures with impaired awareness). Each seizure lasted for 1 minute and then stopped spontaneously. The 4-h video-EEG (VEEG) in the absence of seizures did not capture epileptiform discharge but revealed 2–4 Hz low amplitude slow wave activity in both the awake and sleep states. His cranial MRI showed slightly higher T2 and FLAIR signal in the left parieto-occipital lobe (Figure 1B). Cranial ultrasound revealed subependymal cysts in bilateral ventricles and mild enlargement of bilateral ventricles. Echocardiography detected the patent foramen oval.

At the age of two and a half months, the child suffered from frequent myoclonic seizures and tonic-clonic seizures, more than ten times a day. VEEG revealed the spike-and-slow-wave complex and sharp-wave in the left occipital and temporal regions, and the spike-and-slow-wave complex in the right occipital region during the sleep period. One epileptiform discharge in the left occipital region corresponds to the tonic seizures and tonic-clonic seizures observed in the video. Laboratory tests showed homocysteinemia (HCY) of 22.43 μmol/L, Hb of 98 g/L, mean corpuscular volume (MCV) of 108.1 fL, HCT of 30.50%, albumin of 37.1 g/L, total protein 52.1 g/L, and high neuron-specific enolase (NSE) level of 32.64 ng/mL, indicating hyperhomocysteinemia and macrocytic anemia. Serum vitamin B12, LAC, folate, FBG, thyroid function, and urine metabolism were normal. After treated with oral vigabatrin and pulse adrenocorticotropic hormone (ACTH) therapy, the frequency of seizures were significantly reduced. L-5-methyltetrahydrofolate (L-5-MTHF) and mecobalamin were orally taken regularly, and individualized rehabilitation therapy was carried out.

He still had focal seizures 1–2 times per day and recurrent eczematous dermatitis when he was 5.5 months of age. Topiramate were added orally, and no significant seizures were observed so far. The severe eczematous dermatitis was improved significantly after orally taken zinc gluconate (35 mg, bid) for 2 weeks. Repeat laboratory tests revealed a normal HCY level of 8.59 μmol/L, a slightly low Hb level of 120 g/L and a high NSE level of 39.87 ng/mL. VEEG at eight and a half months of age still showed the spike-and-slow-wave complex and sharp-wave in bilateral occipital and temporal regions. His cranial MRI at 9 months revealed decreased white matter volume, patchy high T2 and FLAIR signals beside the lateral ventricle, indicating cerebral dysplasia (Figure 1B). Other routine examinations and laboratory tests were normal.

At the follow-up of 13 months of age, he could pronounce “mom or dad”, interact with people, exchange objects from one hand to the other, sit without assistance, and stand for 2 min with support. The muscle tension of the extremities was lower than before. His Gesell assessment still revealed a moderate GDD: 57 in gross motor, 50 in fine motor, 46 in adaptability, 46 in language ability, and 57 in social ability.

Family 2

Patient 3 (P3) was a 30-month-old boy admitted to our department due to delayed motor milestone in 2022. He was the only child of a non-consanguineous couple (family 2) and was delivered at term by cesarean section with a normal birth weight (Figure 1A). After birth, he developed pathological jaundice, which was improved after exchange transfusion and phototherapy. He had feeding difficulties, slow growth rate, irritability, and recurrent diarrhea in the neonatal period without any drug or food allergies. The boy had normal motor milestones before 1 year old and could stand without assistance at 14 months of age. But he was unable to walk by himself until he was 30 months old, and even at that point he still showed bad stability and was prone to falling. Besides, he could only speak a few simple words and pronounce unclearly at 30 months. Griffith psychomotor developmental scale showed large motor developmental age of 20.5–21 months, individual-social developmental age of 19.5 months, language developmental age of 26–26.5 months, hand-eye coordinated developmental age of 18–8.5 months, and performance developmental age of 15–15.5 months, indicating GDD. Physical examination showed unstable muscle tone, and involuntary movements. He also had pigeon-toes and limited dorsiflexion of the feet. His EEG result was normal, and cranial MRI revealed partial cortical thickening in bilateral frontoparietal lobes, small bilateral white matter extent, and slightly higher white matter T2 and T2-Flair signals (Figure 1B). No abnormalities were detected in other routine examinations and laboratory tests of periphery blood.

After having regular rehabilitation for 3 months, his walking stability, cognitive level, speech and pronounce, hand function, and dorsiflexion limitation of both feet were improved. The involuntary movements of limbs were reduced. He could run, feet jump, climb 3 to 5 stairs, recognize size and color, distinguish up and down, and sing short nursery rhymes, but still had irritability and a slower response comparing with his peers.

### Genetic and molecular analysis

Through trio-WES analysis and Sanger sequencing, four variants were identified in the MTHFS gene. The pedigree diagrams of family 1 and family 2 were presented in Figure 1A.

**FIGURE 2:**
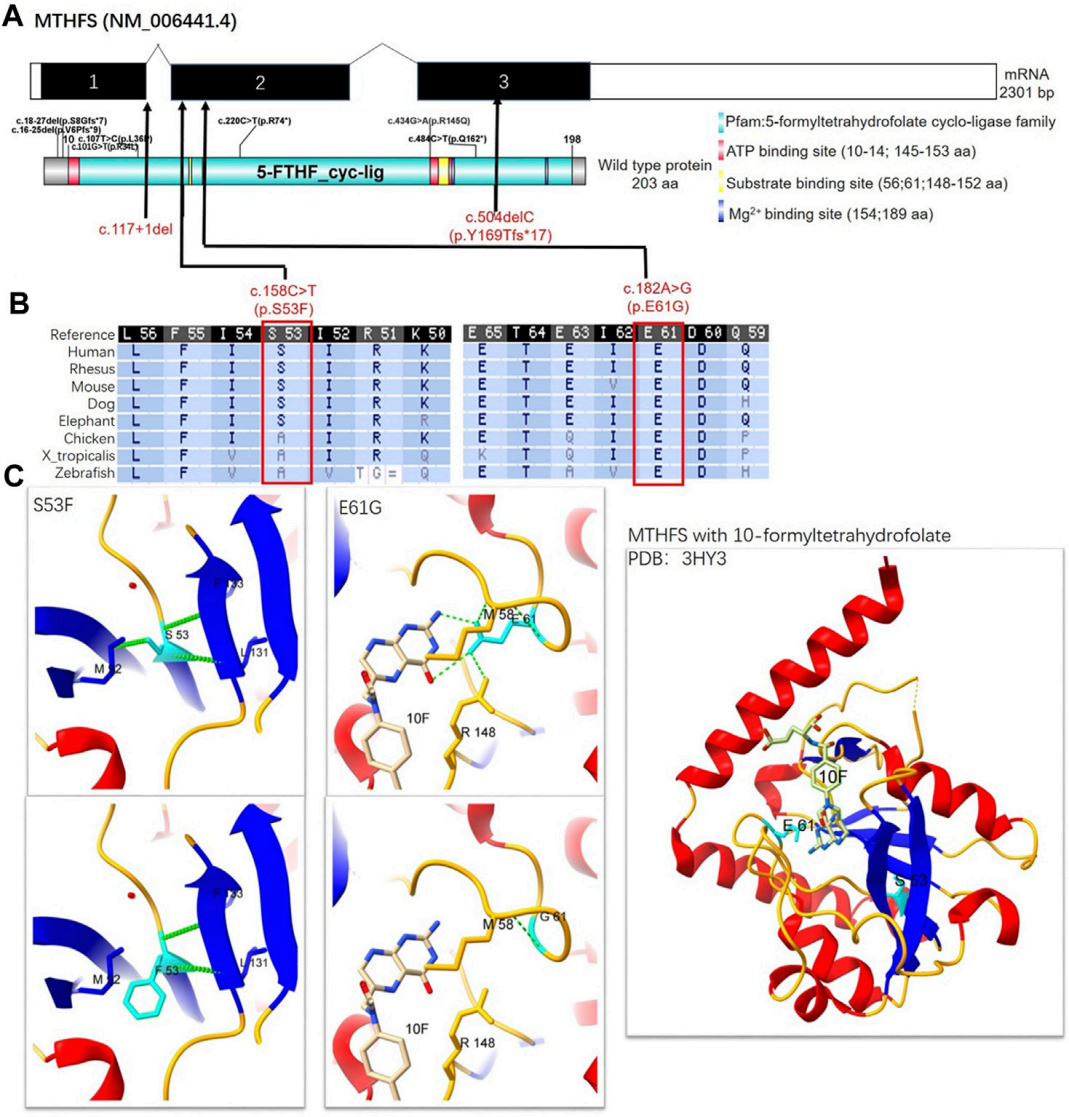
Schematic representation, conservation analysis, and structural analysis of the MTHFS protein. **(A)** The NM_006441.4 transcript encodes a longer isoform NP_006432.1 as the canonical sequence. It has 3 exons and encodes 203-amino-acid protein. MTHFS protein is also known as 5-formyltetrahydrofolate cyclo-ligase (10–198aa) with ATP binding sites (red box), substrate-binding sites (yellow box), and Mg2+ binding sites (dark blue box). Seven variants had been identified in previous published literature (small black text) and four novel variants were identified in this study (red text). **(B)** The conservation of MTHFS: S53 and G61 residues across multiple different species. **(C)** The crystal structure of the human MTHFS with 10-formyltetrahydrofolate is visualized using Protein Data Bank (PDB) structure 3HY3. β-sheet, α-helix, coil, and H-bond are shown in blue, red, orange, and green colors, respectively. S53 contacts with M92, F133, and L131 by hydrogen bonds, while F53 fails to form a hydrogen bond with M92. E61 contacts with M58 and R148 by hydrogen bonds and the carboxyl terminal of E61 also interacts with the NA2 and N3 of the substrate. G61 only contacts with M58. 10F: 10-formyltetrahydrofolate.

To be specific, P1 and P2 carried the same compound heterozygous variation in the MTHFS gene (NM_006441.4) consisting of c.158C>T (p.S53F) and c.504del (p.Y169fs*17), which were inherited from his father and mother, respectively (Figure 1C). The NM_006441.4 transcript encodes NP_006432.1 (isoform a). As a canonical sequence, it has 3 exons and encodes 203-amino-acid protein (Figure 2A). The variant of c.504del (p.Y169fs*17) is a detrimental frameshift variant at exon 3 that is unlikely to initiate the nonsense-mediated mRNA decay (NMD) and predicted to produce an abnormal 184aa product (Figure 2A). The other c.158C>T (p.S53F) variant is located at exon 2 and predicted to be deleterious by Provean, SIFT, PolyPhen-2, Mutation taster, and CADD. Moreover, S53 residue is relatively conserved in multiple species (Figure 2B). To evaluate the potential pathogenicity, we examined the residue change of S53F on the crystal structure of MTHFS protein (Figure 2C). The hydrogen bonds between S53 residue and multiple residues around stabilize the β-sheet structure. Replacement of S53 with phenylalanine would break the bond between S53 and M92 and affect the relative functions (Figure 2C). In addition, according to the ACMG guidelines, c.158C>T (p.S53F) is likely pathogenic (PM2+PM3+PP1+PP3+PP4) and c.504del (p.Y169fs*17) is pathogenic (PVS1+PM2+PP1+PP4). Neither of these variants has been observed or reported previously in any public genomic variants database (such as dbSNP, gnomAD, 1,000 Genomes, ESP6500, ExAC, Colinear, Clinvitae, SWISS, and HGMD).

P3 from family 2 carried a different compound heterozygous variation consisting of c.117+1del and c.182A>G (p.E61G), which were inherited from his father and mother, respectively (Figure 1C). The canonical splice site variant c.117+1del is located at intron 1 (Figure 2A) and is predicted to cause improper splicing that alters the reading frame through exon skipping or intron retention, resulting in a possible NMD or encoding an aberrant protein. Regarding the other variant, multiple *in silico* analysis predictions show that c.182A>G (p.E61G) variant is deleterious. Homogeneous analysis demonstrated that E61 is highly conserved in different species (Figure 2B). In the MTHFS protein model, E61 localizes in the substrate binding site and contacts M58 and R148 by hydrogen bonds. The carboxyl terminal of E61 also interact with the NA2 and N3 of 10-FTHF substrate. The change of E61G would disrupt the hydrogen bonds with the M58 and R148 residues and break the interaction with substrate (Figure 2C). According to the ACMG guidelines, c.117+1del (PVS1+PM2+PP4) and c.182A>G (p.E61G) (PM2+PM3+PP3+PP4) are both likely pathogenic. Neither variant was reported in the public population databases such as dbSNP, 1,000 genomes, and gnomAD.

Combining the clinical characteristics and genetic results, all patients were diagnosed as MTHFS deficiency, and all four variants we identified in the MTHFS gene were novel.

## Discussion

Folate metabolic network provides indispensable cofactors in nucleotide synthesis, neurotransmitter synthesis, methionine regeneration, and amino acid metabolism in several aspects of cell division, methylation, and homeostasis, especially in the CNS. Thus, folate metabolic deficiency can cause a variety of neurological impairments (Zheng and Cantley, 2019). MTHFS deficiency is a kind of inborn errors of folate metabolism caused by mutations in the MTHFS gene. As an extremely rare neurodevelopmental disorder with microcephaly, epilepsy, and hypomyelination (NEDMEHM), the phenotype was first created in OMIM (MIM: #618367) in 2019. The primary signs and symptoms include developmental delay (DD), microcephaly, hypertonia, feeding difficulties, exaggerated startle response or irritability, epilepsy, and cranial nerves affected in early infancy (Rodan et al., 2018; Romero et al., 2019; Sakthivel et al., 2020; Cavusoglu et al., 2022; Vafaee-Shahi et al., 2022).

MTHFS acts as the only enzyme in the folate-mediated one-carbon pathway (FOCM) known to catalyze both endogenous 5-FTHF and exogenous folic acid into 5,10-MTHF, which is subsequently reduced to 5-MTHF and other reduced folates (Dayan et al., 1995). Therefore, the lack of MTHFS (homozygous mutations) results in the increased level of 5-FTHF and the low-normal 5-MTHF level in CSF (Romero et al., 2019; Sakthivel et al., 2020). In some patients with MTHFS deficiency (heterozygous mutations), there is a low folate CSF level (Rodan et al., 2018; Cavusoglu et al., 2022). Several mechanisms could be involved to explain why cerebral folate deficiency contributes to the possible pathophysiology of this neurodevelopmental disorder. First, the downstream low-level 5-MTHF in MTHFS deficiency could lead to a lack of S-adenosylmethionine (SAM)—a methyl donor of the myelin basic protein and then decrease the stability of myelin. Besides, the substituted generation of SAM through choline oxidation pathway leads to CNS choline deficiency, which may trigger aberrant myelination due to a decline in sphingomyelin and phosphatidylcholine levels (Romero et al., 2019). Second, 5-MTHF is the largest contributor to serum folate and participates in various 5-MTHF-dependent biological processes, including neurotransmitter syntheses such as dopamine, serotonin, and norepinephrine (Miller, 2008). Third, the increased 5-FTHF level may hinder serine hydroxy methyltransferase and 5-aminoimidazole-4-carboxamide ribonucleotide formyltransferase activity, inhibit thymidylate synthesis and *de novo* purine metabolism, and impair DNA synthesis in cell growth and division of processes such as red blood cell production, therefore contribute to hyperhomocysteinemia and anemia (Field et al., 2006). High homocysteine level would attenuate the integrity and increase permeability of blood-brain barrier (Kamath et al., 2006; Beard et al., 2011), lead to toxin-mediated brain injury-induced seizures (Mattson and Shea, 2003; Vitvitsky et al., 2004). Fourth, MTHFS deficiency also exhibits an intramitochondrial folate deficiency, which would shunt intramitochondrial translation of mitochondrial methionyl-tRNA and lead to secondary mitochondrial dysfunction, resulting in deficient energy for brain development (Bertrand et al., 1995; Fox and Stover, 2008; Wang et al., 2021).

We summarized clinical features of the three new cases with MTHFS deficiency and previously published ones (Table 1). First described in 2018, Rodan et al. reported two unrelated patients, aged 8 and 11 years, who both carried biallelic variants in the MTHFS gene and showed similar phenotypes. Microcephaly, feeding difficulties, exaggerated startle response, short stature, severe GDD, progressive spasticity and hypomyelination were initially found in early infancy. Laboratory test detected low-normal levels of 5-MTHF in the CSF. The onset of epilepsy occurs when the patient was 2–3 years of age (Rodan et al., 2018). Next in 2019, Romero et al. reported the third patient of MTHFS deficiency with the identical presentation, such as microcephaly, short stature, spasticity, hypertonia, DD, cerebral hypomyelination and seizures. Novel findings of high-arched palate, macrocytic anemia, and elevated neopterin were recorded (Romero et al., 2019). Then, Sakthivel et al. described the fourth patient with homozygous mutation in the MTHFS gene in 2020. The patient suffered from seizures since she was 3 years old, and she had vocal fold paralysis at 9 years old. Dysphagia with feeding difficulties, hypertonia, DD, decreased level of CSF 5-MTHF were shown. Unfortunately, the patient passed away at the age of 18 due to hepatic failure. However, this study did not present the neuroimaging results (Sakthivel et al., 2020). The fifth patient with MTHFS deficiency is a girl characterized by similar features, including microcephaly, GDD, hypertonia, seizure, and cerebral hypomyelination, while she was initially diagnosed with homocystinuria. Genetic analysis helped to confirm the diagnosis (Cavusoglu et al., 2022). The sixth case was mainly characterized by microcephaly, DD, seizures, high signal intensity in the bilateral parietal and occipital lobes, macrocytic anemia, increased homocysteine, and low CSF folate level. Her unusual manifestations included nystagmus, hypotonia, and bilateral deep brain calcification. Genetic tests identified novel variants in the MTHFS gene and confirmed the diagnosis of MTHFS deficiency (Vafaee-Shahi et al., 2022).

**TABLE 1:** Clinical features and therapy response of the patients with MTHFS variants.

No	Sex	Age	Mutation type	Variants	Brain MRI	Seizure types/EEG	Developmental abnormalities	Laboratory studies	Therapy/response	References
1	M	16 m	Com het	c.504del (p.Y169Tfs*17) c.158C>T (p.S53F)	Widened bilateral frontotemporal subarachnoid space, decreased white matter volume, small corpus callosum, slightly enlarged lateral ventricles, widened sulcus fissure, and abnormal myelination	Fever-induced seizures, including partial seizures, tonic seizures, tonic-clonic seizures, atypical absence seizures, status epilepticus, petit mal seizures. Generalized epileptiform discharges	DD, short stature, low weight, broad nasal bridge, long nasolabial fold, tent-shaped upper lip, athetosis	Anemia, Low HCT; Normal serum vitamin B12, folate, thyroid function, urine metabolism	Oxcarbazepine, VPA & LTG for epilepsy, but seizures continued, SUDEP at 22 months of age	Present study
2	M	3 m	Com het	c.504del (p.Y169Tfs*17) c.158C>T (p.S53F)	Widened bilateral sulcus fissure of the cortex, widened bilateral frontotemporal subdural space, decreased volume of white matter, lateral paraventricular slightly high T2 and FLAIR signal	Myoclonic seizures and tonic-clonic seizures. Multifocal spike-and-slow-wave complex, sharp wave, bilateral posterior head obviously	Poor head control and eye contact and interaction, microcephaly, oxycephaly, long nasolabial fold, tent-shaped upper lip, high arched palate, eczematous dermatitis	Macrocytic anemia, Low HCT, Hyperhomocysteinemia; Normal serum vitamin B12, folate, thyroid function, urine metabolism	VGB & TPM for epilepsy, seizure stopped 6 weeks later; VitB12 & L-5-MTHF, rehabilitation, cognitive and motor abilities improved	Present study
3	M	2.5y	Com het	c.182A>G (p.E61G) c.117+1delG	Decreased white matter volume	None	DD, feeding difficulties, slow growth rate, irritability, and recurrent diarrhea	Normal	Comprehensive rehabilitation therapy, cognitive and motor abilities improved	Present study
4	M	8y	Com het	c.107T>C (p.L36P) c.434G>A (p.R145Q)	Delayed myelination, mild lateral ventriculomegaly, and cerebellar fissure widening with a lack of expected growth of the corpus callosum	Generalized slowing, absence of posterior dominant rhythm, and biposterior polyspike-wave discharges	DD, short stature, microcephaly, feeding difficulties, exaggerated startle response	Normal initial CSF & CSF plasma ratios	Oral L-5-MTHF & IM increase CSF 5-MTHF levels and mild improvements in functioning	Rodan et al. (2018)
5	M	11y	Com het	c.434G>A (p.R145Q) c.484C>T (p.Q162X)	Hypomyelination, prominence of the cerebellar fissures	Atonic, gelastic and tonic seizures. Slowing and generalized epileptiform discharges	DD, limited vocabulary and articulation difficulties; microcephaly, thick eyebrows, full cheeks, epiblepharon, tapered fingers; feeding difficulties, exaggerated startle response, diffuse hypertonia	Normal CSF lactate, protein, glucose, cell count, amino acids, and neurotransmitter metabolites	DR (lacosamide & LTG, rufinamide, LEV, & CZP)	Rodan et al. (2018)
6	F	11 m	Homo	c.220C>T (p.R74X)	The abnormal white matter with under-myelination of the internal capsules, relative under-myelination of the remainder of the subcortical white matter, and a thin corpus callosum	Periodic lateralized epileptiform discharges	DD, Low weight, short stature, microcephaly, strabismus, high arched palate, diffuse hypertonia, spasticity	Macrocytic anemia, elevated CSF neopterin, decreased 5-MTHF	Seizures controlled with LEV, and baclofen for spasticity	Romero et al. (2019)
7	F	18y	Homo	c.101G>T (p.R34L)	NA	Acute repetitive cluster seizures	DD, vocal fold paralysis; poor feeding and dysphagia; generalized hypertonia, epileptic aphasia; central visual and auditory processing disorder; other complications	Low CSF 5-MTHF	Zarontin for seizures, L-5-MTHF for low CSF 5-MTHF. Died of hepatic failure at 18 years of ages	Sakthivel et al. (2020)
8	F	7.5y	Homo	c.18_27del (p.S8Gfs*7)	Vermis hypoplasia, cerebellar and cortical atrophy, increased signals in white matter dominantly in the deep and subcortical area in favor of hypomyelination. Putamen involvement similar to metal sedimentation (hyperintensity in T1 and hypo-intensity in T2)	Fever-induced seizure at 18 months of age	DD, microcephaly, diffuse spastic tonicity in extremities, increased deep tendon reflexes	Homocystinuria; Normal evaluation of metabolic disorders (tandem mass spectrometry, acylcarnitine profile, urine organic acid, serum ammonia, lactate, pyruvate)	Intramuscular vitamin B12 injection, vitamin B6, Biotin, folinic acid, and vitamin C	Vafaee-Shahi et al. (2022)
9	F	27 m	Homo	c.16_25del (p.V6Pfs*9)	Restricted diffusion in the bilateral putamen, globus pallidus, parietal and occipital lobes; calcification of the bilateral putamen, globus pallidus, caudate nucleus, and parietal and occipital lobes	Intractable seizures; a focal spike and slow waves in the left parietal region	DD, short in stature, low weight, microcephaly, strabismus, hypertelorism, broad nasal bridge, malar hypoplasia, prominent nasolabial folds, short tent-shaped mouth, irritability, feeding difficulties, hypotonia, nystagmus	Macrocytic anemia, increased homocysteine, low CSF folate; Normal serum vitamin B12, folate, thyroid function	Seizures controlled with LEV, valproate, TPM & CZP	Cavusoglui et al. (2022)

Note: Age at testing; MRI, magnetic resonance imaging; EEG, electroencephalogram; F, female; M, male; ms, months; ys, years; Com het, Compound heterozygous; Homo, Homozygous; NA, not available; DD, developmental delay; HCT, hematocrit; CSF, cerebrospinal fluid; 5-MTHF, 5-methyltetrahydrofolate; VPA, valproic acid; LEV, levetiracetam; TPM, topiramate; CZP, clonazepam; DR, drug-resistant; LTG, lamotrigine; NZP, nitrazepam; IM, intramuscular methylcobalamin; L-5-MTHF, L-5-methyltetrahydrofolate; SUDEP, sudden unexpected death in epilepsy.

In this study, we present three more cases with MTHFS deficiency from two unrelated families, with phenotypes varying from mild to severe. Similarly, three patients all showed DD and cerebral hypomyelination. P1 and P2 were siblings both had some craniofacial dysmorphisms including ocular hypertelorism, broad and flat nasal bridge, long nasolabial groove, and tent-shaped upper lip. Apart from GDD, they also suffered from seizures and anemia. P1 was the first case of SUDEP and P2 had severe seizures at birth, indicating a more severe phenotype than other cases. While P3 had feeding difficulty, irritability, and mild DD without seizures. Unlike the previously recorded cases, P3 had poor muscle coordination and more involuntary movements, which was easily misdiagnosed in clinical practice as dyskinetic cerebral palsy. Due to the mild disease presentation of P3, we suppose that the c.117+1delG variant only generates partial splicing errors, resulting in an incomplete loss of MTHFS activity. Although previous research found that the amino acid change of E61A almost completely abolished catalytic activity of MTHFS (Wu et al., 2009), the outcome of c.182A>G (p.E61G) remains unknown. Further experiments are warranted to interpret the pathogenic variants in order to elucidate the genetic heterogeneity of MTHFS deficiency.

Moreover, the athetosis of P1, the eczematous dermatitis of P2, and the recurrent diarrhea of P3 were novel features reported in MTHFS deficiency. Athetosis might be related to neurological impairments induced by folate metabolic deficiency, as mentioned above. In terms of the eczema, studies have shown that a lower methyl donor pool (e.g., lower serum folate levels, impaired folate metabolism) is associated with a higher risk of atopy (Friso et al., 2002; Husemoen et al., 2006). Since the folate metabolic deficiency might lead to epigenetic changes that tilt the immunophenotypic balance toward allergic diseases (Matsui and Matsui, 2009). Considering the mechanism of the link between MTHFS deficiency and recurrent diarrhea, regeneration of the intestinal surface may depend on adequate folate status on the one hand, and may be related to the role of folate in innate and/or adaptive antigen-specific immune responses on the other (Dhur et al., 1991; Manger et al., 2011). Disturbance of folate metabolism disrupts the normal renewal of intestinal epithelial cells or interferes with the immune response, prolonging recovery from the initial infection and leading to recurring episodes of diarrhea. The above manifestations were only seen in a few individuals; thus, more research is still needed to explore the specific mechanisms.

So far, there is no definitive treatment protocol for MTHFS deficiency (Table 1). Treatment with folinic acid and folic acid in a patient with low-normal CSF 5-MTHF level was unsuccessful, indicating that the non-reduced form of folate has no improvement in the management of cerebral folate deficiency (Rodan et al., 2018). When treated with L-5-MTHF, a useable reduced form of folate, and mecobalamin, a methylated form of cobalamin to utilize 5-MTHF, the motor functions and CSF 5-MTHF level improved (Rodan et al., 2018; Ramaekers and Quadros, 2022). Since most of the patients had seizures, anti-epilepsy therapy is also the main treatment. Previous study showed that one MTHFS deficiency patient with refractory seizures and recurrent episodes of hyperthermia responded to lamotrigine (Rodan et al., 2018). However, in our cases, P1 had fever-induced, refractory, status seizures, and his seizures still continued with the treatment of oxcarbazepine, sodium valproate, and lamotrigine.

Fortunately, P2, the younger brother of P1, had myoclonic seizures and tonic-clonic seizures stopped, and his HCY level returned to normal with a combination of anti-epilepsy drugs (vigabatrin and topiramate), L-5-MTHF, and mecobalamin. The severe eczema was also improved by taking zinc gluconate orally. Combined with goal-targeted rehabilitation, P2’s cognitive and motor abilities had become better than before. Till now, P3 has not shown any signs of seizures, but a longer-term follow-up with repeat MRI and EEG is required. It is worth noting that severe complications are the leading cause of death in this disease. One case was reported to die at the age of 18 due to hepatic failure (Sakthivel et al., 2020), and P1 died at 22 months old because of SUDEP. So, regular follow-up and meticulous care are essential in the management of such disease.

MTHFS deficiency can present with varying degrees of neurodevelopmental abnormalities, ranging from mild GDD to refractory epilepsy to SUDEP. A broad range of differential diagnosis may exist between it and other diseases, such as cerebral folate transport deficiency (FOLR1), dihydrofolate reductase (DHFR) deficiency, methylenetetrahydrofolate reductase (MTHFR) deficiency, hereditary folate malabsorption (SLC46A1), *etc.* Thus, genetic analysis is required to confirm the diagnosis of MTHFS deficiency. Furthermore, the mother of P1 and P2 had recurrent pregnancy loss (RPL), which could be linked to a folate metabolism disorder during embryonic development (Hong Li and Marren, 2018). There is mounting evidence that impaired FOCM and elevated homocysteine levels are associated with adverse pregnancy outcomes (Rady, 2018; Mo et al., 2019; Zhang et al., 2019; Liu et al., 2020; Nwogu et al., 2020; Bala et al., 2021). As folate levels fall, dTMP synthesis and DNA methylation levels were decreased, resulting in unstable genomes and aneuploid chromosomal abnormalities (Wang et al., 2004). The elevated HCY may lead to placental arterial embolization, impaired vascular endothelium and coagulation, and cause placental insufficiency with direct toxic effects on the embryo (Eskes, 2001; Jakubowski, 2019; Zaric et al., 2019). Therefore, RPL is introduced as a danger sign in MTHFS deficiency, and prenatal genetic screening on the MTHFS gene in such families would avoid inborn errors of metabolism.

In conclusion, we reported three new cases of MTHFS deficiency and the process of diagnosis and treatment, which expands the phenotype spectrum with the novel features of athetosis, eczema, and recurrent diarrhea. Meanwhile, we identified four novel variants in the MTHFS gene and expanded the genotype spectrum of this rare disease. Further studies on exploring the genotype-phenotype correlation of MTHFS deficiency are warranted.

## Data Availability

All data generated or analyzed during this study are included in this published article. The original contributions presented in this study are publicly available. The MTHFS variants NM_006441.4: c.158C>T (p.S53F), c.504del (p.Y169Tfs*17), c.117+1del and c.182A>G (p.E61G) were submitted to the LOVD database (https://databases.lovd.nl/shared/variants/MTHFS/), with the LOVD Variant ID: https://databases.lovd.nl/shared/variants/0000933017#00014020, https://databases.lovd.nl/shared/variants/0000933018#00014020, https://databases.lovd.nl/shared/variants/0000933019#00014020, https://databases.lovd.nl/shared/variants/0000933020#00014020.
